# Relationship between Maternal Body Mass Index and Obstetric and Perinatal Complications

**DOI:** 10.3390/jcm9030707

**Published:** 2020-03-05

**Authors:** Ana Ballesta-Castillejos, Juan Gómez-Salgado, Julián Rodríguez-Almagro, Inmaculada Ortiz-Esquinas, Antonio Hernández-Martínez

**Affiliations:** 1 Department of Obstetrics and Gynecology, Talavera de la Reina, 45600 Toledo, Spain; ana.ballesta81@gmail.com; 2 Department of Sociology, Social Work and Public Health, University of Huelva, 21071 Huelva, Spain; salgado@uhu.es; 3 Safety and Health Posgrade Program, Universidad Espíritu Santo, Guayaquil 091650, Ecuador; 4 Department of Nursing, Faculty of Nursing of Ciudad Real, University of Castilla-La Mancha, 13071 Ciudad Real, Spain; antomatron@gmail.com; 5 Department of Obstetrics and Gynaecology, Alcázar de San Juan,13600 Ciudad Real, Spain; inmaores@hotmail.com

**Keywords:** body mass index, obstetric complications, public health

## Abstract

Over the past few decades, overweight and obesity have become a growing health problem of particular concern for women of reproductive age as obesity in pregnancy has been associated with increased risk of obstetric and neonatal complications. The objective of this study is to describe the incidence of obstetric and perinatal complications in relation to maternal body mass index (BMI) at the time prior to delivery within the Spanish Health System. For this purpose, a cross-sectional observational study was conducted aimed at women who have been mothers between 2013 and 2018 in Spain. Data were collected through an online survey of 42 items that was distributed through lactation associations and postpartum support groups. A total of 5871 women answered the survey, with a mean age of 33.9 years (SD = 4.26 years). In the data analysis, crude odds ratios (OR) and adjusted odds ratios (AOR) were calculated through a multivariate analysis. A linear relationship was observed between the highest BMI figures and the highest risk of cephalopelvic disproportion (AOR of 1.79 for obesity type III (95% CI: 1.06–3.02)), preeclampsia (AOR of 6.86 for obesity type III (3.01–15.40)), labor induction (AOR of 1.78 for obesity type III (95% CI: 1.16–2.74)), emergency C-section (AOR of 2.92 for obesity type III (95% CI: 1.68–5.08)), morbidity composite in childbirth (AOR of 3.64 for obesity type III (95% CI: 2.13–6.24)), and macrosomia (AOR of 6.06 for obesity type III (95% CI: 3.17–11.60)), as compared with women with normoweight. Women with a higher BMI are more likely to develop complications during childbirth and macrosomia.

## 1. Introduction

Over the past few decades, overweight and obesity have become a growing health problem in the Western world [1], so much so that WHO now considers it one of the most relevant health problems [2]. The distribution of this problem is not homogeneous among the population, being women’s rates higher than men’s [3], and this makes obesity a problem that greatly affects women of reproductive age.

Maternal obesity has emerged globally as one of the main obstetric challenges [4,5], whose prevalence increases linearly not only in developed countries, but also in developing countries [6]. At European level, more than 30% of pregnant women are obese, and the prevalence of overweight women reaches 50% (2).

Many experimental and epidemiological studies show that nutritional changes in the prenatal and postnatal stages of life can have a significant impact on the child’s health and development [2,3,7,8,9]. Maternal overweight, obesity, and morbid obesity are already known to be associated with adverse obstetric and neonatal outcomes [3,10]. In particular, maternal obesity is associated with gestational diabetes mellitus (GDM) [11,12], preeclampsia [12], congenital malformations and macrosomia [3], preterm birth [3], low Apgar scores [4], admittance to neonatal intensive care unit [13], low-weight newborns [3,14], and with an increase in obstetric interventions such as pregnancy caesarean section [12,15] and labor induction [16,17]. In the long term, maternal obesity is associated with an increased risk of diabetes mellitus and cardiovascular diseases in women, and with an increased risk of childhood obesity in the newborn [2,4]. Postnatally, obese women are less likely to successfully breastfeed [18,19], tend to have longer hospital stays, and are at an increased risk of postnatal infections [16].

In Spain, data indicate that the average population weight has increased since records exist and, if this trend continues [20], obesity rates will have increased by 16% by 2030 [21]. Despite this, we do not currently have studies that relate these data to obstetric and perinatal results. Moreover, the evaluated results have been studied based on pregestational obesity, with weight gain being highly variable among pregnant women. For these reasons, we consider that it would be relevant to describe the incidence of obstetric and perinatal complications in relation to maternal body mass index (BMI) at the time prior to delivery within the Spanish Health System.

## 2. Material and Methods

### 2.1. Design and Selection of Study Subjects

This cross-sectional observational study was aimed at women who have been mothers between 2013 and 2018 in Spain. This study has received the approval of the Ethics and Clinical Research Committee of Alcázar de San Juan, in Spain, with protocol number 92-C.

The participants were women over the age of 18, who understood Spanish, and who agreed to fill in the questionnaire. Before doing so, the participants had to read an information sheet about the purpose of the study and give their consent to participate in it. After this, they were provided with the necessary information to be able to complete the questionnaire. Participants could voluntarily provide an e-mail address or phone number through which they would be contacted in case any additional information related to the study was needed.

For the sample size estimation, the maximum modelling criterion, that requires 10 events for each independent variable to be included in the multivariate model, has been used [22]. The main event used for this calculation was the emergency C-section rate, located in other studies at around 27% [23]. Considering a minimum of 15 independent variables, a minimum of 150 women with emergency C-section would be required, representing a minimum total population of 555 women under study within this scenario.

For data collection, an anonymous online questionnaire was designed with 42 items (36 yes/no questions and 6 multiple answer questions) on sociodemographic variables, obstetric variables, and complications during pregnancy, delivery, and postpartum (Supplementary material). The questionnaire was disseminated among the Spanish lactation and postpartum support associations and groups. Those responsible for these groups were in charge of disseminating the said questionnaire among their members.

The variables included in the study were as follows:

The main independent variable: body mass index (calculated by dividing kilograms of weight by the squared height in meters), categorized into normoweight (18.5–24.9), overweight (25–29.9), obesity grade I (30.0–34.5), obesity grade II (35.0–39.9), and obesity grade III (>40.0) [4,5].

The independent adjustment variables were maternal age, income level, level of education, tobacco consumption, assistance to maternal education, number of births, twin pregnancy, preeclampsia during pregnancy, diet-controlled gestational diabetes, insulin-controlled gestational diabetes, hyperthyroidism, hypothyroidism, anemia, intrahepatic cholestasis, risk of preterm birth, deep vein thrombosis, oligoamines, polyhydramnios, altered fetal heart rate (FHR) during delivery, stained amniotic fluid (AF), vaginal bleeding, uterine rupture, nonprogression of delivery, cephalopelvic disproportion, intrapartum fever, intrapartum preeclampsia, labor induction, end of delivery, tearing, low weight (2500 g), macrosomia (>4000 g), newborn admission, postpartum-related subsequent maternal surgery, maternal admission into intensive care unit, and maternal readmission.

In addition, four “composite” morbidity variables were created: pregnancy (including preeclampsia during pregnancy, diet-controlled gestational diabetes, insulin-controlled gestational diabetes, hyperthyroidism, hypothyroidism, anemia, intrahepatic cholestasis, risk of preterm birth, deep vein thrombosis); delivery (including altered fetal heart rate (FHR) during delivery, stained amniotic fluid (AF), vaginal bleeding, nonprogression of delivery, cephalopelvic disproportion, intrapartum fever, intrapartum preeclampsia, labor induction, end of delivery by caesarean, and severe tearing (type III-IV)); neonatal (including prematurity, low weight (<2500 g), macrosomia (>4000 g), and maternal admission to intensive care unit); and postpartum (including maternal postpartum surgery related to the delivery, maternal admission to intensive care unit, and maternal readmission).

### 2.2. Statistical Analysis Used

First, a descriptive analysis was performed using absolute and relative frequencies for categorical variables. Then, a bivariate analysis was performed between the main sociodemographic, clinical characteristics, and complications regarding the BMI during pregnancy, childbirth, and postpartum, using the Pearson’s chi-squared test. Then, the crude odds ratios (OR) and adjusted odds ratios (AOR) were calculated with their respective 95% confidence intervals (95% CI) to determine the relationship between the BMI and the obstetric and neonatal complications. To do this, a multivariate analysis was performed by means of binary logistic regression and multinomial logistic regression (for the birth-type variable). Regarding collinearity treatment, the authors opted for an automatic forward and backward stepwise regression. Therefore, although the whole set of variables was included in each analysis, those predictors which did not show a statistical relationship were progressively eliminated, thus reducing the risk of collinearity.

All analyses were performed using the SPSS v24.0 statistical package.

## 3. Results

The studied population was 5871 women. Of these, 27.30% (*n* = 1607) had normoweight, 46.40% (*n* = 2722) were overweight, 19.60 (*n* = 1153) had obesity type I, 5.00% (*n* = 293) obesity type II, and 0.13% (*n* = 96) obesity type III.

A bivariate analysis was then performed to determine the sociodemographic, obstetric, neonatal, and admittance characteristics in relation to the BMI at the time of delivery.

Regarding the relationship between the BMI and the most important sociodemographic characteristics, we found a statistical association with maternal age, academic level, family income level, smoking habit, and attendance to childbirth and maternity classes. However, we did not find any statistically significant differences with respect to nationality. All the details of this analysis can be found in Table 1.

When analyzing obstetric factors in relation to BMI, a relationship was found between BMI and the number of births, twin pregnancy, prematurity, hypertensive pregnancy disorders, gestational diabetes, both insulin-controlled and diet-controlled, risk of preterm birth, deep vein thrombosis, oligoamines, and polyhydramnios (Table 2).

In the next step, a multivariate analysis was carried out in which all the variables that could potentially be related to BMI were incorporated. Regarding the variables related to complications during childbirth, we observed a linear trend in five of them (Table 3) among women with different degrees of obesity as compared with women with normoweight: cephalopelvic disproportion, preeclampsia, induced delivery, emergency C-section and Composite morbidity delivery.

Finally, in the composite morbidity birth variable, an AOR of 1.47 (95% CI: 1.29–1.68) was obtained for overweight women, an AOR of 2.00 (95% CI: 1.69–2.37) in the case of obesity type I, AOR 3.51 (95% CI: 2.55–4.83) for women with obesity type II, and an AOR of 3.64 (95% IQ: 2.13–6.24) in the case of obesity type III.

The same process was followed with those variables related to maternal and fetal complications during the postpartum, finding a linear relationship with the presence of macrosomia. All the details of this analysis can be found in Table 4.

## 4. Discussion

In this study, we have analyzed the relationship between BMI and the incidence of obstetric and perinatal complications. While multiple associations have been identified, we consider those where a clear linear trend has been observed as more relevant. Specifically, an association was found between higher figures of BMI and pelvic-cephalic disproportion, preeclampsia, induced delivery, and emergency C-section. As for the newborn, we have described a relationship between maternal BMI and higher rates of macrosomia.

In several studies, obesity or maternal overweight have been linked to complications during pregnancy, such as hypertensive disorders or macrosomia [24], that may increase the risk of C-section [1,25]. Regarding the data from our study, the increase in emergency C-section rates may be related not only to the factors previously indicated, but also to the increase in altered fetal heart rate frequency and the higher rate of cephalopelvic disproportion in this population. However, some studies point to abnormalities in uterine contractility and an increase in fat deposits in the soft areas of the pelvis [26]. This increase in C-section rates has already been described in other studies [1,10,24,25,27] and may also influence the risk of neonatal complications and the possibilities of newborn admission.

In this sense, the adjustment of maternal BMI would reduce induction rates, the risk of an emergency caesarean, and neonatal admissions. Thus, this improvement would not only imply health-related aspects, but also an important reduction of healthcare expenses.

The higher incidence of newborns with macrosomia in this group of mothers has been explained by the hypothesis proposed by Pedersen [28], who suggested that increased glucose concentrations in the diabetic mother leads to hyperglycemia and fetal hyperinsulinemia that causes higher fetal growth [26]. In short, after birth, these newborns are more likely to suffer from hypoglycemia [29] and hospital admissions [30].

On the other hand, macrosomia is an independent risk factor of child obesity [31,32]. This fact is especially relevant as macrosomia is seen as an intermediate factor in the relationship between maternal obesity and child obesity. This way, all the efforts aimed at adjusting maternal BMI would imply a positive improvement regarding both maternal and child health.

For all this, Spain, where obesity rates have progressively increased until reaching about 18.4% [33] of the population, may greatly benefit from implementing prevention programs and encouraging women access to preconception consultations so as to reach the optimum BMI prior to gestation.

### Strengths and Limitations

As for the study limitations, an information bias is acknowledged as data was collected using self-reported questionnaires which surveyed the participants’ perception. In order to reduce the confounding factors, the design of the questionnaire did not require a high level of education to be completed. In this regard, the questions were asked in a basic and simple way, and any participant could understand them, regardless of their level of education. It is not possible to completely rule out the confusion bias inherent in observational studies, but it has been attempted to be controlled by using multivariate analysis techniques.

Among the main strengths of the study, we can highlight the large sample size and the scarcity of studies carried out in women assisted by the Spanish Health System. On the other hand, the prevalence figures for obesity are in line with other studies, such as the one by Parveen et al. [4], in which a prevalence of obesity of 23.2% was found. The relationship between excessive maternal weight and adverse obstetric and neonatal outcomes has already been researched. Most of the previous studies took pregestational BMI into account and gestational weight gain above established standards during pregnancy [34]. Instead, we have used weight at birth as a criterion, thus encompassing previous weight and total gestational weight gain. However, although BMI measurement prior to delivery provides a broader view of weight gain during pregnancy, it also introduces a bias since weight gain may be multicausal and an amalgamation of pre-pregnancy weight, gestational weight gain, and the fetus’ weight.

For all the previously described aspects and, especially, due to the cross-sectional nature of the study, casual inference is limited in this case. However, there are shared outcomes with those from other authors’ research which highlight the negative impact of maternal obesity on obstetric and perinatal results.

## 5. Conclusions

Therefore, we consider that excessive BMI at the time of delivery implies an independent risk of increased maternal and fetal morbidity. It is for this reason that the professionals responsible for the surveillance of mothers must have an impact on proper weight gain during pregnancy and, especially, in the pregestational stage. Health education covering healthy lifestyles is a key element for women to have a low-risk pregnancy and delivery.

## Figures and Tables

**Table 1:** Sociodemographic and obstetric characteristics of the women according to the body mass index (BMI).

Variable	BMI					*p* Value *
	Normoweight *n* (%)	Overweight *n* (%)	Obesity Type I n (%)	Obesity Type II *n* (%)	Obesity Type III *n* (%)	
**Maternal age**						<0.001
<25 years	38 (2.4)	60 (2.2)	34 (2.9)	10 (3.4)	7 (7.3)	
25–35	938 (58.4)	1545 (56.8)	697 (60.5)	192 (65.5)	66 (68.8)	
>35 years	631 (39.3)	1117 (41.0)	422 (36.6)	91 (31.1)	23 (24.0)	
**Level of education**						<0.001
None	0 (0.0)	4 (0.1)	2 (0.2)	2 (0.7)	0 (0.0)	
Primary	28 (1.7)	48 (1.8)	21 (1.8)	6 (2.0)	3 (3.1)	
Secondary	340 (21.2)	715 (26.3)	379 (32.9)	132 (45.1)	45 (46.9)	
University	1239 (77.1)	1955 (71.8)	751 (65.1)	153 (52.2)	48 (50.0)	
**Monthly family income (euros)**						<0.001
<1000	81 (5.0)	139 (5.1)	74 (6.4)	30 (10.2)	9 (9.4)	
1000–2000	469 (29.2)	891 (32.7)	416 (36.1)	128 (43.7)	45 (46.9)	
2000–3000	571 (35.5)	924 (33.9)	397 (34.4)	83 (28.3)	29 (30.2)	
3000–4000	331 (20.6)	533 (19.6)	202 (17.5)	41 (14.0)	9 (9.4)	
>4000	155 (9.6)	235 (8.6)	64 (5.6)	11 (3.8)	4 (4.2)	
**Nationality**						0.861
Spanish	1508 (93.8)	2557 (93.9)	1076 (93.3)	276 (94.2)	92 (95.8)	
Foreign	99 (6.2)	165 (6.1)	77 (6.7)	17 (5.8)	4 (4.2)	
**Childbirth and maternity classes**						0.017
No	375 (23.3)	602 (22.1)	271 (23.5)	61 (20.8)	35 (36.5)	
Yes	1232 (76.7)	2120 (77.9)	882 (76.5)	232 (79.2)	61 (63.5)	
**Pre-pregnancy smoking habits**						<0.001
No	1296 (80.6)	2104 (77.3)	834 (72.3)	201 (68.6)	69 (71.9)	
Yes	311 (19.4)	618 (22.7)	319 (27.7)	92 (31.4)	27 (28.1)	

* Pearson’s χ^2^ test.

**Table 2:** Obstetric characteristics of the women according to the BMI.

Variable	BMI					*p* Value *
	Normoweight *n* (%)	Overweight *n* (%)	Obesity Type I *n* (%)	Obesity Type II *n* (%)	Obesity Type III *n* (%)	
**Number of pregnancies**						0.058
One	860 (53.5)	1456 (53.5)	611 (53.0)	149 (50.9)	50 (52.1)	
Two	562 (35.0)	910 (33.4)	356 (30.9)	99 (33.8)	31 (32.3)	
Three	137 (8.5)	232 (8.5)	120 (10.4)	33 (11.3)	11 (11.5)	
Four	36 (2.2)	84 (3.1)	39 (3.4)	8 (2.7)	2 (2.1)	
Five or more	12 (0.7)	40 (1.5)	27 (2.3)	4 (1.4)	2 (2.1)	
**Number of deliveries**						**<0.001**
None	259 (16.1)	595 (21.9)	311 (27.0)	98 (33.4)	33 (34.4)	
One	869 (54.1)	1391 (51.1)	542 (47.0)	132 (45.1)	40 (41.7)	
Two	422 (26.3)	645 (23.7)	261 (22.6)	50 (17.1)	20 (20.8)	
Three	48 (3.0)	80 (2.9)	33 (2.9)	10 (3.4)	3 (3.1)	
Four	9 (0.6)	10 (0.4)	4 (0.3)	1 (0.3)	0 (0.0)	
Five or more	0 (0.0)	1 (0.0)	2 (0.2)	2 (0.7)	0 (0.0)	
**Twin pregnancy**						**0.045**
No	1583 (98.5)	2652 (97.4)	1118 (97.0)	284 (96.9)	92 (95.8)	
Yes	24 (1.5)	70 (26)	35 (3.0)	9 (3.1)	4 (4.2)	
**Prematurity**						**0.011**
>37	1472 (91.6)	2557 (93.9)	1065 (92.4)	278 (94.9)	93 (96.9)	
<37	135 (8.4)	165 (6.1)	88 (7.6)	15 (5.1)	3 (3.1)	
**Hypertensive states**						**<0.001**
No	1550 (96.5)	2568 (94.3)	1032 (89.5)	239 (81.6)	77 (80.2)	
Yes	57 (3.5)	154 (5.7)	121 (10.5)	54 (18.4)	19 (19.8)	
**Gestational diabetes-diet**						**<0.001**
No	1429 (88.9)	2463 (90.5)	988 (85.7)	223 (76.1)	75 (78.1)	
Yes	178 (11.1)	259 (9.5)	165 (14.3)	70 (23.9)	21 (21.9)	
**Gestational diabetes-insulin**						**<0.001**
No	1575 (98.0)	2660 (97.7)	1124 (97.5)	275 (93.9)	89 (92.7)	
Yes	32 (2.0)	62 (2.3)	29 (2.5)	18 (6.1)	7 (7.3)	
**Hyperthyroidism**						0.292
No	1528 (95.1)	2601 (95.6)	1100 (95.4)	272 (92.8)	90 (93.8)	
Yes	79 (4.9)	121 (4.4)	53 (4.6)	21 (7.2)	6 (6.3)	
**Hypothyroidism**						0.457
No	1398 (87.0)	2375 (87.3)	984 (85.3)	251 (85.7)	86 (89.6)	
Yes	209 (13.0)	347 (12.7)	169 (14.7)	42 (14.3)	10 (10.4)	
**Anemia**						0.056
No	967 (60.2)	1605 (59.0)	732 (63.5)	186 (63.5)	63 (65.6)	
Yes	640 (39.8)	1117 (41.0)	421 (36.5)	107 (36.5)	33 (34.4)	
**Intrahepatic cholestasis**						0.794
No	1584 (98.6)	2691 (98.9)	1137 (98.6)	291 (99.3)	95 (99.0)	
Yes	23 (1.4)	31 (1.1)	16 (1.4)	2 (0.7)	1 (1.0)	
**Risk of preterm birth**						**<0.001**
No	1433 (89.2)	2529 (92.9)	1062 (92.1)	276 (94.2)	87 (90.6)	
Yes	174 (10.8)	193 (7.1)	91 (7.9)	17 (5.8)	9 (9.4)	
**Deep vein thrombosis**						**0.042**
No	1580 (98.3)	2695 (99.0)	1138 (98.7)	289 (98.6)	92 (95.8)	
Yes	27 (1.7)	27 (1.0)	15 (1.3)	4 (1.4)	4 (4.2)	
**Oligoamines**						**0.018**
No	1561 (97.1)	2618 (96.2)	1092 (94.7)	285 (97.3)	93 (96.9)	
Yes	46 (2.9)	104 (3.8)	61 (5.3)	8 (2.7)	3 (3.1)	
**Polyhydramnios**						**0.001**
No	1502 (93.5)	2480 (91.1)	1037 (89.9)	259 (88.4)	82 (85.4)	
Yes	105 (6.5)	242 (8.9)	116 (10.1)	34 (11.6)	14 (14.6)	
**Composite pregnancy morbidity**						**0.088**
No	621 (38.6)	1051 (38.6)	419 (36.3)	93 (31.7)	31 (32.3)	
Yes	986 (61.4)	1671 (61.4)	734 (63.7)	200 (68.3)	65 (67.7)	

* Pearson’s χ^2^ test.

**Table 3:** Complications during delivery according to the BMI.

Variable	BMI				
	Normoweight *n* (%)	Overweight *n* (%)	Obesity Type I *n* (%)	Obesity Type II *n* (%)	Obesity Type III *n* (%)
**Altered FHR**					
No	1451 (90.3)	2379 (87.4)	989 (85.8)	248 (84.6)	82 (85.4)
Yes	156 (9.7)	343 (12.6)	164 (14.2)	45 (15.4)	62 (14.6)
OR CI 95%	[1]	**1.34 (1.10–1.64)**	**1.54 (1.22–1.95)**	**1.69 (1.18–2.41)**	1.59 (0.88–2.87)
^a^ AOR CI 95%	[1]	**1.36 (1.11–1.67)**	**1.54 (1.21–1.96)**	**1.62 (1.12–2.35)**	1.60 (0.87–2.95)
**Stained AF**					
No	1540 (95.8)	2545 (93.5)	1067 (92.5)	274 (93.5)	82 (85.4)
Yes	67 (4.2)	177 (6.5)	86 (7.5)	19 (6.5)	14 (14.6)
OR CI 95%	[1]	**1.60 (1.20–2.13)**	**1.85 (1.33–2.57)**	1.59 (0.94–2.70)	**3.92 (2.12–7.28)**
^a^ AOR CI 95%	[1]	**1.57 (1.17–2.10)**	**1.81 (1.30–2.53)**	1.53 (0.89–2.62)	**4.01 (2.13–7.58)**
**Vaginal bleeding**					
No	1530 (95.2)	2587 (95.0)	1087 (94.3)	273 (93.2)	89 (92.7)
Yes	77 (4.8)	135 (5.0)	66 (5.7)	20 (6.8)	7 (7.3)
OR CI 95%	[1]	1.04 (0.78–1.38)	1.21 (0.86–1.69)	1.46 (0.88–2.42)	1.56 (0.70–3.49)
^b^ AOR CI 95%	[1]	1.07 (0.80–1.66)	1.16 (0.82–1.66)	1.33 (0.78–2.28)	1.58 (0.69–3.63)
**Uterine rupture**					
No	1595 (99.3)	2708 (99.5)	1148 (99.6)	288 (98.3)	95 (99.0)
Yes	12 (0.7)	14 (0.5)	5 (0.4)	5 (1.7)	1 (1.0)
OR CI 95%	[1]	0.69 (0.32–1.49)	0.58 (0.20–1.65)	2.31 (0.81–6.60)	1.40 (0.18–10.87)
^b^ AOR CI 95%	[1]	0.52 (0.24–1.16)	0.38 (0.13–1.16)	1.21 (0.39–3.68)	0.67 (0.08–5.55)
**Nonprogression of delivery**					
No	1349 (83.9)	2205 (81.0)	893 (77.5)	223 (76.1)	75 (78.1)
Yes	258 (16.1)	517 (19.0)	260 (22.5)	70 (23.9)	21 (21.9)
R CI 95%	[1]	**1.23 (1.04–1.44)**	**1.52 (1.26–1.84)**	**1.64 (1.22–2.22)**	1.46 (0.89–2.42)
^a^ AOR CI 95%	[1]	1.09 (0.92–1.30)	**1.26 (1.02–1.55)**	1.24 (0.89–1.71)	1.03 (0.89–1.71)
**Cephalopelvic disproportion**					
No	1424 (88.6)	2303 (84.6)	935 (81.1)	234 (79.9)	74 (77.1)
Yes	183 (11.4)	419 (15.4)	218 (18.9)	59 (20.1)	22 (22.9)
OR CI 95%	[1]	**1.42 (1.18–1.71)**	**1.81 (1.47–2.25)**	**1.96 (1.42–2.71)**	**2.31 (1.10–3.82)**
^a^ AOR CI 95%	[1]	**1.35 (1.11–1.63)**	**1.59 (1.28–1.99)**	**1.62 (1.15–2.29)**	**1.79 (1.06–3.02)**
**Fever**					
No	1540 (95.8)	2574 (94.6)	1081 (93.8)	275 (93.9)	84 (87.5)
Yes	67 (4.2)	148 (5.4)	72 (6.2)	18 (6.1)	12 (12.5)
R CI 95%	[1]	1.33 (0.98–1.77)	**1.53 (1.09–2.15)**	1.50 (0.88–2.57)	**3.28 (1.71–6.31)**
^b^ AOR CI 95%	[1]	1.23 (0.91–1.67)	**1.39 (1.97–1.98)**	1.31 (0.75–2.31)	**2.58 (1.29–5.17)**
**Preeclampsia**					
No	1577 (98.1)	2654 (97.5)	1103 (95.7)	266 (90.8)	85 (88.5)
Yes	30 (1.9)	68 (2.5)	50 (4.3)	27 (9.2)	11 (11.5)
OR CI 95%	[1]	1.35 (0.87–2.08)	**2.38 (1.51–3.77)**	**5.34 (3.12–9.12)**	**6.80 (3.30–14.04)**
^a^ AOR CI 95%	[1]	1.46 (0.93–2.31)	**1.98 (1.22–3.23)**	**4.15 (2.31–7.47)**	**6.86 (3.01–15.40)**
**Labor induction**					
No	1121 (69.8)	1708 (62.7)	688 (59.7)	151 (51.5)	52 (54.2)
Yes	486 (30.2)	1014 (37.3)	465 (40.3)	142 (48.5)	44 (45.8)
OR CI 95%	[1]	**1.37 (1.20–1.56)**	**1.56 (1.33–1.83)**	**2.17 (1.69–2.79)**	**1.95 (1.29–2.96)**
AOR CI 95%	[1]	**1.37 (1.20–1.57)**	**1.44 (1.22–1.70)**	**1.94 (1.49–2.53)**	**1.78 (1.16–2.74)**
**Mode of delivery**					
Vaginal	1075 (66.9)	1629 (59.8)	639 (55.4)	136 (46.4)	47 (49.0)
Instrumental	264 (16.4)	449 (16.5)	176 (15.3)	46 (15.7)	14 (14.6)
OR CI 95%	[1]	1.12 (0.95–1.33)	1.12 (0.91–1.39)	1.38 (0.96–1.98)	1.21 (0.66–2.24)
^c^ AOR CI 95%	[1]	1.09 (0.91–1.30)	1.04 (0.93–1.31)	1.27 (0.86–1.86)	1.11 (0.58–2.11)
Planned caesarean	89 (5.5)	220 (8.1)	108 (9.4)	41 (14.0)	6 (6.3)
OR CI 95%	[1]	**1.63 (1.26–2.11)**	**2.04 (1.52–2.75)**	**3.64 (2.42–5.49)**	1.54 (0.64–3.71)
AOR CI 95%	[1]	1.41 (1.06–1.86)	1.72 (1.24–2.38)	2.83 (1.78–4.50)	1.09 (0.42–2.79
Emergency caesarean	179 (11.1)	424 (15.6)	230 (19.9)	70 (23.9)	29 (30.2)
OR CI 95%	[1]	**1.56 (1.29–1.89)**	**2.16 (1.74–2.69)**	**3.09 (2.23–4.30)**	**3.71 (2.27–6.04)**
AOR CI 95%	[1]	1.44 (1.17–1.76)	**1.89 (1.49–2.40)**	**2.49 (1.73–3.59)**	**2.92 (1.68–5.08)**
**III-IV tearing**					
No	1589 (98.9)	2689 (98.8)	1136 (98.5)	284 (96.9)	95 (99.0)
Yes	18 1.1	33 (1.2)	17 (1.5)	9 (3.1)	1 (1.0)
OR CI 95%	[1]	1.08 (0.61–1.93)	1.32 (0.68–2.57)	**2.80 (1.24–6.29)**	0.93 (0.12–7.04)
^d^ AOR CI 95%	[1]	1.06 (0.59–1.91)	1.34 (0.67–2.66)	**3.04 (1.30–7.15)**	1.04 (0.13–8.11)
**Composite morbidity delivery**					
No	728 (45.3)	995 (36.6)	336 (29.1)	57 (19.5)	18 (18.8)
Yes	879 (54.7)	1727 (63.4)	817 (70.9)	236 (80.5)	78 (81.3)
OR CI 95%	[1]	**1.44 (1.27–1.63)**	**2.01 (1.72–2.37)**	**3.43 (2.53–4.65)**	**3.59 (2.13–6.05)**
^e^ AOR CI 95%	[1]	**1.47 (1.29–1.68)**	**2.00 (1.69–2.37)**	**3.51 (2.55–4.83)**	**3.64 (2.13–6.24)**

All the results were adjusted by the following variables: Maternal age, nulliparity, twin pregnancy, maternity training, previous C-section, newborn weight, economic income, level of education, nationality, composite pregnancy morbidity, and smoking habit. ^a^ Complementary adjusted by the following: induced delivery, epidural analgesia, and gestational age. ^b^ Complementary adjusted by the following: induced delivery, episiotomy, severe tearing, and type of delivery. ^c^ Complementary adjusted by the following: induced delivery, and epidural analgesia. ^d^ Complementary adjusted by the following: episiotomy, and type of delivery. ^e^ Complementary adjusted by the following: composite pregnancy morbidity. FHR, fetal heart rate; AF, amniotic fluid; OR, odds ratio; AOR, adjusted odds ratio. Bold means statistically significant.

**Table 4:** Postpartum maternal/fetal complications according to the BMI.

Variable	BMI				
	Normoweight *n* (%)	Overweight *n* (%)	Obesity Type I *n* (%)	Obesity Type II *n* (%)	Obesity Type III *n* (%)
**Prematurity**					
No	1472 (91.6)	2557 (93.9)	1065 (92.4)	278 (94.9)	93 (96.9)
Yes	135 (8.4)	165 (6.1)	88 (7.6)	15 (5.1)	3 (3.1)
OR CI 95%	[1]	**0.70 (0.56–0.89)**	0.90 (0.68–1.19)	0.59 (0.34–1.02)	0.35 (0.11–1.13)
AOR CI 95%	[1]	**0.67 (0.53–0.86)**	0.79 (0.59–1.06)	**0.49 (0.28–0.87)**	**0.27 (0.08–0.89)**
**Low weight**					
No	1488 (92.7)	2560 (94.3)	1076 (93.4)	271 (93.1)	95 (99.0)
Yes	118 (7.3)	155 (5.7)	76 (6.6)	20 (6.9)	1 (1.0)
OR CI 95%	[1]	**0.76 (0.60–0.98)**	0.89 (0.66–1.20)	0.93 (0.57–1.52)	**0.13 (0.02–0.96)**
^a^ AOR CI 95%	[1]	0.74 (0.55–1.01)	**0.67 (0.55–0.97)**	0.88 (0.47–1.58)	**0.10 (0.02–0.96)**
**Macrosomia**					
No	1561 (97.2)	2608 (96.1)	1072 (93.1)	267 (91.8)	81 (84.4)
Yes	45 (2.8)	107 (3.9)	80 (6.9)	24 (8.2)	15 (15.6)
OR CI 95%	[1]	1.42 (0.99–2.03)	**2.59 (1.78–3.76)**	**3.12 (1.87–5.20)**	**6.24 (3.44–12.01)**
^a^ AOR CI 95%	[1]	1.32 (0.93–1.89)	**2.51 (1.72–3.8)**	**2.75 (1.62–4.68)**	**6.06 (3.17–11.60)**
**Newborn admission**					
No	1422 (88.5)	2399 (88.1)	991 (85.9)	231 (78.8)	82 (85.4)
Yes	185 (11.5)	323 (11.9)	162 (14.1)	62 (21.2)	14 (14.6)
OR CI 95%	[1]	1.04 (0.85–1.25)	**1.26 (1.00–1.58)**	**2.06 (1.50–2.84)**	1.31 (0.73–2.36)
^b^ AOR CI 95%	[1]	1.17 (0.95–1.46)	**1.33 (1.03–1.72)**	**2.55 (1.78–3.65)**	1.75 (0.93–3.31)
**Postpartum surgery related to delivery**					
No	1551 (96.5)	2634 (96.8)	1105 (95.8)	280 (95.6)	90 (93.8)
Yes	56 (3.5)	88 (3.2)	48 (4.2)	13 (4.4)	6 (6.3)
OR CI 95%	[1]	0.93 (0.66–1.30)	1.20 (0.81–1.78)	1.29 (0.69–2.38)	1.85 (0.78–4.40)
^c^ AOR CI 95%	[1]	0.85 (0.60–1.20)	1.01 (0.68–1.52)	0.98 (0.52–1.85)	1.51 (0.62–3.68)
**Postpartum maternal ICU admission**					
No	1575 (98.0)	2681 (98.5)	1128 (97.8)	285 (97.3)	94 (97.9)
Yes	32 (2.0)	41 (1.5)	25 (2.2)	8 (2.7)	2 (2.1)
OR CI 95%	[1]	0.75 (0.47–1.20)	1.09 (0.64–1.85)	1.38 (0.63–3.03)	1.05 (0.25–4.44)
^c^ AOR CI 95%	[1]	0.69 (0.43–1.12)	0.81 (0.47–1.41)	0.98 (0.44–2.22)	0.84 (0.19–3.65)
**Readmission after discharge**					
No	1580 (98.3)	2666 (97.9)	1132 (98.2)	281 (95.9)	94 (97.9)
Yes	27 (1.7)	56 (2.1)	21 (1.8)	12 (4.1)	2 (2.1)
OR CI 95%	[1]	1.23 (0.77–1.95)	1.09 (0.61–1.93)	**2.50 (1.25–4.99)**	1.25 (0.29–5.32)
^c^ AOR CI 95%	[1]	1.16 (0.72–1.85)	0.94 (0.53–1.69)	1.98 (0.97–4.06)	1.02 (0.24–4.40)
**Composite postpartum morbidity**					
No	1507 (93.8)	2558 (94.0)	1071 (92.9)	265 (90.4)	86 (89.6)
Yes	100 (6.2)	164 (6.0)	82 (7.1)	28 (9.6)	10 (10.4)
OR CI 95%	[1]	0.97 (0.75–1.30)	1.15 (0.85–1.56)	**1.59 (1.03–2.47)**	1.75 (0.88–3.48)
^d^ AOR CI 95%	[1]	0.90 (0.69–1.17)	0.96 (0.70–1.31)	1.22 (0.78–1.93)	1.45 (0.72–2.93)

All the results were adjusted by the following variables: Maternal age, nulliparity, twin pregnancy, maternity training, previous caesarean, economic income, level of education, nationality, composite pregnancy morbidity, and smoking habit. ^a^ Complementary adjusted by the following: prematurity. ^b^ Complementary adjusted by the following: type of delivery, altered FHR, and stained amniotic fluid. ^c^ Complementary adjusted by the following: severe tearing, type of delivery, pregnancy complications, preeclampsia, vaginal bleeding during delivery, fever, and uterine rupture. ^d^ Complementary adjusted by the following: composite pregnancy morbidity, and composite delivery morbidity. Bold means statistically significant.

## Supplementary Materials

The following are available online at https://www.mdpi.com/2077-0383/9/3/707/s1, QUESTIONNAIRE.
