# Supplementary material for: Relationship between Maternal Body Mass Index and Obstetric and Perinatal Complications
Source: J Clin Med. 2020 Mar 5;9(3):707. doi: 10.3390/jcm9030707 (PMC7141254; doi:10.3390/jcm9030707)
Supplement: Supplementary file 1 [file jcm-09-00707-s001.pdf]

Supplemental Material: QUESTIONNAIRE

1. What is your age? (in years)
< 25 years
25-35 years
>35 years

2. What is your level of studies?
No studies
Primary
Secondary
University

3. Have you smoked before pregnancy?
No
Yes

4. Monthly family income (in euros)
<1000
1000-2000
2000-3000
3000-4000
>4000

5. Did you attend maternal education for your previous pregnancy?
No
Yes

6. What is your nationality?
Spanish
Foreign

7. How many pregnancies have you had?
One
Two
Three
Four
Five or more

8. How many vaginal deliveries have you had?
None
One
Two
Three
Four
Five or more

Now, we will ask you about your last pregnancy and delivery

Important: You may only answer if the pathology has been diagnosed by a health professional and appears in your medical record.

9. How many weeks were you pregnant when you gave birth?
Term (37 or more weeks)
Preterm (less than 37 weeks)

10. Was this a twin pregnancy?
No
Yes

11. During your last pregnancy, did you suffer from diagnosed high blood pressure?
No
Yes

12. During your last pregnancy, were you diagnosed with diabetes (high sugar levels) that was treated with diet?
No
Yes

13 During your last pregnancy, were you diagnosed with diabetes (high sugar levels) that was treated with insulin?
No
Yes

14. During your last pregnancy, were you diagnosed with hyperthyroidism?
No
Yes

15. During your last pregnancy, were you diagnosed with hypothyroidism?
No
Yes

16. During your last pregnancy, were you diagnosed with anaemia that was treated with iron?
No
Yes

17. During your last pregnancy, were you diagnosed with a liver disease (Intrahepatic cholestasis)?
No
Yes

18. During your last pregnancy, were you diagnosed with premature delivery (preterm) risk?
No
Yes

19. During your last pregnancy, were you diagnosed with venous thrombosis?
No
Yes

20. During your last pregnancy, were you diagnosed with low levels of amniotic fluid?
No
Yes

21. During your last pregnancy, were you diagnosed with high levels of amniotic fluid?
No
Yes

22. Did you suffer any other complication during pregnancy?
No
Yes: State:____

23. During your last delivery, did you have any problem due to diagnosed uterine rupture?
No
Yes

24. During your last delivery, did you have any problem due to fever, as assessed by a physician?
No
Yes

25. During your last delivery, did you have diagnosed high blood pressure (preeclampsia)?
No
Yes

26. Was it induced delivery?
No
Yes

27. How did your delivery end?
It was a normal or eutocic delivery
It was a forceps-aided delivery
It was a planned caesarean
It was an emergency caesarean

28. Did you have an episiotomy performed (a cut made by the midwife or the gynaecologist on the perineum during the delivery)?
No
Yes

29. Did you suffer any perineal tearing (other than episiotomy) that required suturing/stitches?
No
Yes
Yes, and later an appointment at hospital was scheduled for this reason (in this case, this would be a degree III-IV tearing, as these imply subsequent consultation follow-up).

30. Did you suffer any other complication during the delivery?
No
Yes: State:____

31. Could you have skin-to-skin contact with your baby after the delivery?
No
Yes

32. Did you start maternal breastfeeding in the first hour following the delivery?
No

Yes

33. During your last pregnancy, did you give birth to a baby with weight < 2500 grams?
No
Yes

34. During your last pregnancy, did you give birth to a baby with weight > 4000 grams?
No
Yes

35. Was your child admitted to hospital ?
No
Yes

36. At discharge from hospital, was the newborn exclusively breastfed?
No
Yes

37. After the delivery, did you undergo any delivery-related surgery?
No
Yes

38. After the delivery, were you admitted at the ICU?
No
Yes

39. After discharge, were you admitted to hospital again?
No
Yes

40. Did you suffer any complication during the post-partum?
No
Yes: State:____

41. What was your weight (in kg) at the moment of delivery? (State weight as measured by the health professional during the consultation)

42. What is your height (in cm)? (State height as measured by the health professional during the consultation)
